# Analysis of influencing factors of cognitive frailty in older adults community patients based on restricted cubic spline

**DOI:** 10.3389/fpubh.2025.1666043

**Published:** 2025-10-29

**Authors:** Shuai Chen, Jiahe Chen, Shuzhi Peng

**Affiliations:** 1 Department of Respiratory and Critical Care Medicine, Funing People’s Hospital, Yancheng, China; 2 College of Health Management, Shanghai Jian Qiao University, Shanghai, China

**Keywords:** cognitive frailty, older adults, restricted cubic spline, depression, sleep quality, community health

## Abstract

**Objective:**

To investigate the influencing factors of cognitive frailty in older adults community-dwelling patients and analyze the nonlinear relationships between key variables such as age, depression scores, sleep quality, and cognitive frailty, providing a basis for accurately identifying high-risk populations and developing individualized intervention strategies.

**Method:**

A simple random sampling method was employed to select 16 community health service centers across 16 districts in Shanghai, conducting questionnaire surveys among 1,692 older adults patients with multiple coexisting chronic conditions. The restricted cubic spline (RCS) model was used to analyze the dose–response relationship between age, depression score (CES-D), sleep quality (PSQI), and cognitive frailty, while controlling for confounding factors such as gender, types of chronic diseases, and social engagement.

**Results:**

The detection rate of cognitive frailty was 44.56%. RCS analysis revealed significant nonlinear associations between age, depression score, sleep quality, and cognitive frailty. Key inflection points where the risk of cognitive frailty significantly increased were age ≥75 years, depression score ≥20 points, and sleep quality score ≤5 points. After adjusting for confounding factors, the nonlinear relationship between depression score and cognitive frailty remained significant (*p* = 0.043), while the associations with age and sleep quality tended to be linear.

**Conclusion:**

Cognitive frailty is relatively common among community-dwelling older adults individuals, with age, depression, and sleep quality being its significant influencing factors. The restricted cubic spline model effectively reveals the nonlinear interaction characteristics of these factors, providing a scientific basis for implementing stratified early warning and precise interventions at the community level.

## 1 Introduction

Cognitive frailty, as an important concept in the field of geriatrics, generally refers to the co-occurrence of cognitive impairment and physical frailty in older adults, though the two components may not always present simultaneously or with equal severity (1). This phenomenon not only increases the risks of dementia, falls, and disability, but also significantly impacts quality of life and independent living ability. Recent studies have shown that the prevalence of cognitive frailty in the older adults population is as high as 10–20%, particularly more common among those aged 75 and above (2). Its pathological mechanisms involve multifactorial interactions including neurodegeneration, inflammatory responses, and metabolic disorders. In clinical practice, early identification and intervention for cognitive frailty are crucial. For example, multimodal strategies such as cognitive training, nutritional support, and physical exercise can effectively delay progression and reduce the incidence of complications (3). Furthermore, cognitive frailty, as a key indicator for predicting health outcomes in the older adults, has been incorporated into international geriatric guidelines, promoting the development and application of relevant screening tools and intervention programs (4). Research indicates that the improvement of community and family support systems, such as regular health check-ups and psychological counseling, has a significant effect on the prevention and control of cognitive decline (5). Through comprehensive interventions, older adults individuals can not only maintain cognitive function but also improve their quality of life and reduce the burden on medical resources (6). In the future, interdisciplinary collaboration and the promotion of personalized programs will be key strategies for addressing cognitive frailty (7). At the same time, strengthening public education and policy support to enhance societal awareness of cognitive decline helps build a more older adults-friendly living environment and further optimizes the overall health status of older adults (8).

The restricted cubic spline model can flexibly capture the complex nonlinear association between continuous variables and cognitive frailty, avoiding potential estimation bias in effect size caused by traditional linear assumptions (9). Compared to traditional statistical models, restricted cubic splines demonstrate unique advantages when handling continuous risk factors (such as age, depression scores, etc.), as they can more accurately reflect dose–response relationships (10). This approach is particularly suitable for complex phenotypes like cognitive frailty, which are influenced by multifactorial nonlinear interactions (11). This study focuses on the older adults population in communities, aiming to leverage the flexibility of this model to systematically explore the association patterns between various bio-psycho-social factors and the occurrence and progression of cognitive frailty, identify key thresholds and sensitive change intervals, and provide evidence-based support for establishing community-level graded early warning criteria and targeted intervention pathways (12, 13). Through in-depth analysis of the functional characteristics of influencing factors, this study is expected to address the gaps in existing literature regarding the dynamic trajectories of risk factors and critical effect values, thereby advancing cognitive frailty prevention and control strategies toward more refined and individualized approaches.

Domestic and international research has identified a series of biomedical, psychosocial, and behavioral risk factors for cognitive frailty in older adults (14–16). However, significant gaps remain in characterizing the dynamic evolution trajectories and nonlinear features of dose–response relationships, particularly the lack of systematic quantitative analysis on critical turning points and threshold effect values of risk factors. This study employs a restrictive cubic spline model to thoroughly analyze the complex nonlinear association patterns and dynamic evolution mechanisms between risk factors and the occurrence and progression of cognitive frailty, thereby providing more timely and targeted evidence-based decision-making support for the stratified management and individualized prevention of cognitive frailty in the older adults community population (see Table 1).

**Table 1:** Comparison of basic characteristics between cognitive frailty group and non-cognitive frailty group (*n* = 1,692).

Variable	Number (*n* = 1,692)	Cognitive frailty (*n* = 938)	Non-cognitive frailty (*n* = 754)	Statistics	***p***
Age, *M* (*Q*₁, *Q*₃)	71. (66, 78)	70 (65, 77)	73 (66, 79)	*Z* = −4.78	<0.001
Depression scores, *M* (*Q*₁, *Q*₃)	18 (12, 27)	16 (11, 25)	21(13, 29)	*Z* = −8.56	<0.001
PSQI scores, *M* (*Q*₁, *Q*₃)	11 (7, 15)	9 (5, 13)	13 (9, 17)	*Z* = −13.74	<0.001
Sex, *n* (%)				*χ*^2^ = 445.08	<0.001
Male	721 (42.61)	613 (65.35)	108 (14.32)		
Female	971 (57.39)	325 (34.65)	646 (85.68)		
BMI, *n* (%)				*χ*^2^ = 1.99	0.369
<18.5	404 (23.88)	213 (22.71)	191 (25.33)		
18.5–24.0	671 (39.66)	372 (39.66)	299 (39.66)		
≥24	617 (36.47)	353 (37.63)	264 (35.01)		
Marital status, *n* (%)				*χ*^2^ = 0.16	0.924
Married	684 (40.43)	378 (40.30)	306 (40.58)		
Unmarried	477 (28.19)	262 (27.93)	215 (28.51)		
Divorce or widowhood	531 (31.38)	298 (31.77)	233 (30.90)		
Education, *n* (%)				*χ*^2^ = 0.32	0.852
Below high school level	768 (45.39)	429 (45.74)	339 (44.96)		
High school	491 (29.02)	274 (29.21)	217 (28.78)		
University level or above	433 (25.59)	235 (25.05)	198 (26.26)		
Residential status, *n* (%)				*χ*^2^ = 0.55	0.760
Living alone	413 (24.41)	223 (23.77)	190 (25.20)		
With family	838 (49.53)	471 (50.21)	367 (48.67)		
With other caregivers	441 (26.06)	244 (26.01)	197 (26.13)		
Types of chronic diseases, *n* (%)				*χ*^2^ = 175.92	<0.001
2–3	428 (25.30)	354 (37.74)	74 (9.81)		
4–6	530 (31.32)	261 (27.83)	269 (35.68)		
7–9	734 (43.38)	323 (34.43)	411 (54.51)		
Smoking status, *n* (%)				*χ*^2^ = 1.46	0.483
No smoking	984 (58.16)	557 (59.38)	427 (56.63)		
Smoking	516 (30.50)	280 (29.85)	236 (31.30)		
Quit smoking	192 (11.35)	101 (10.77)	91 (12.07)		
Drinking, *n* (%)				*χ*^2^ = 5.82	0.055
No drinking	1,127 (66.65)	618 (65.88)	509 (67.60)		
Drinking	374 (22.12)	225 (23.99)	149 (19.79)		
Quit drinking	190 (11.24)	95 (10.13)	95 (12.62)		
Exercise, *n* (%)				*χ*^2^ = 4.94	0.294
Everyday	119 (7.03)	75 (8.00)	44 (5.84)		
6–7 times/week	346 (20.45)	188 (20.04)	158 (20.95)		
4–5 times/week	468 (27.66)	254 (27.08)	214 (28.38)		
2–3 times/week	706 (41.73)	387 (41.26)	319 (42.31)		
<times/week	53 (3.13)	34 (3.62)	19 (2.52)		
Social situation, *n* (%)				*χ*^2^ = 76.74	<0.001
Everyday	52 (3.07)	30 (3.20)	22 (2.92)		
6–7 times/week	392 (23.17)	273 (29.10)	119 (15.78)		
4–5 times/week	304 (17.97)	175 (18.66)	129 (17.11)		
2–3 times/week	508 (30.02)	287 (30.60)	221 (29.31)		
<times/week	436 (25.77)	173 (18.44)	263 (34.88)		

*Z*, Mann–Whitney test; *χ*^2^, Chi-square test; *M*, median; *Q*₁, 1st quartile; *Q*₃, 3rd quartile.

## 2 Objects and methods

### 2.1 Survey subjects

This study adopted a simple random sampling method, selecting one community health service center from each of Shanghai’s 16 districts as research sites to conduct face-to-face questionnaire surveys with outpatients suffering from multimorbidity (The investigation was based on the International Classification of Diseases, 10th Revision (ICD-10), covering over 40 chronic diseases including chronic ischemic heart disease, hypertension, diabetes mellitus, chronic cerebrovascular disease, chronic obstructive pulmonary disease, chronic gastritis, malignant tumors, and Parkinson’s syndrome. Individuals diagnosed with two or more chronic diseases were included in the scope of multimorbidity (17).)

### 2.2 Sample size calculation

The minimum sample size calculation in this study was based on 20 times the number of variables, with 12 variables included and a 10% bias rate taken into account (18), resulting in a calculated minimum sample size of 267. Inclusion criteria: (1) Meeting the diagnostic criteria for chronic diseases in the 10th Revision of the International Classification of Diseases (ICD-10) (17), with a confirmed diagnosis for over 1 year; (2) Clear thinking, willingness to cooperate with this survey and signing an informed consent form; (3) No communication barriers; (4) Age ≥60 years old. Exclusion criteria: (1) Patients with terminal malignant tumors, severe organ dysfunction, or pulmonary incompetence; (2) Those suffering from mental disorders or having cognitive disorders; (3) Severe visual and hearing disorders, dementia, or refusal to participate.

### 2.3 Methods

#### 2.3.1 Survey instruments

(1) General information questionnaire, mainly including gender, age, BMI, marital status, education level, disease type, living situation, smoking and drinking status, and exercise habits.
(2) The clinical and social epidemiological depression inventory (CES-D), developed by Radloff in 1977, is a depression assessment tool designed to evaluate depressive symptoms over a week, with a focus on depressive mood or affect. The scale contains 20 items using a 4-point rating system (0–3), with items 4, 8, 12, and 16 being reverse-scored. A total score ≤9 indicates no depression, 10–16 represents mild depression, 17–24 moderate depression, and >24 indicates moderate-to-severe depression, where higher scores indicate more severe depression. The scale demonstrates good validity across different cultural contexts (19).
(3) The Pittsburgh sleep quality index (PSQI), a widely used tool for assessing sleep disorders, evaluates participants ‘sleep quality over the past month. Comprising seven dimensions and 18 items with a total score of 21 points, a PSQI score ≥8 indicates sleep disorders, where higher scores reflect poorer sleep quality. The Cronbach’s *α* coefficient ranges from 0.832 across dimensions and 0.845 between items (20).

#### 2.3.2 Cognitive frailty assessment tools

Cognitive frailty was defined as the coexistence of physical frailty (FP ≥ 3 points) and cognitive dysfunction (CDR = 0.5 points and MoCA<26 points), excluding Alzheimer’s disease and other types of dementia, according to IANA and IAGG 2013 guidelines (21).

(1) Frailty phenotype (FP): evaluates five frailty symptoms in the target *Homo sapiens* group: decreased walking speed, weight loss, fatigue, reduced grip strength, and inability to walk forward. Each symptom is scored 1 point (0 if absent). Total score 0: no frailty; 1–2: pre-frailty; 3–5: frailty (22). Cronbach’s *α* = 0.897, KMO = 0.890, *p* < 0.05.
(2) Montreal cognitive assessment (MoCA): this scale consists of eight sections designed for cognitive screening in the target *Homo sapiens* population. The total score is 30 points, with ≥26 indicating normal cognition and <26 suggesting the presence of cognitive dysfunction. Its Cronbach’s alpha coefficient and KMO value are 0.839 and 0.895 respectively, with a *p*-value less than 0.05 (23).
(3) The clinical dementia rating (CDR) is used to assess the severity of dementia, comprising six memory items and five other items. Scores: 0 = healthy, 0.5 = questionable dementia, 1 = mild dementia, 2 = moderate dementia, 3 = severe dementia (24). Reliability: Cronbach’s *α* = 0.890, KMO = 0.898, *p* < 0.05.

#### 2.3.3 Data collection methods

Prior to the implementation of this study, approval and cooperation were first obtained from the directors of relevant community outpatient departments. Uniformly trained and qualified investigators distributed paper questionnaires to patients on-site in outpatient settings, using standardized instructions to clarify the survey’s purpose. For chronic disease patients with limited literacy, visual impairments, or difficulties comprehending questionnaire items, investigators maintained a neutral stance to provide explanations, allowing patients to verbally indicate their choices. Upon immediate collection of the questionnaires, investigators promptly checked the completeness and logical consistency of the responses, reminding participants to supplement or correct any inaccurately or incompletely filled items on the spot.

A total of 1,700 questionnaires were distributed in this study. After verification, 8 invalid questionnaires with convergent responses, inaccurate content and logical contradictions were excluded, and finally 1,692 valid questionnaires were obtained, with a valid recovery rate of 99.53% (1,692/1,700).

### 2.4 Statistical analysis

**Figure 1:**
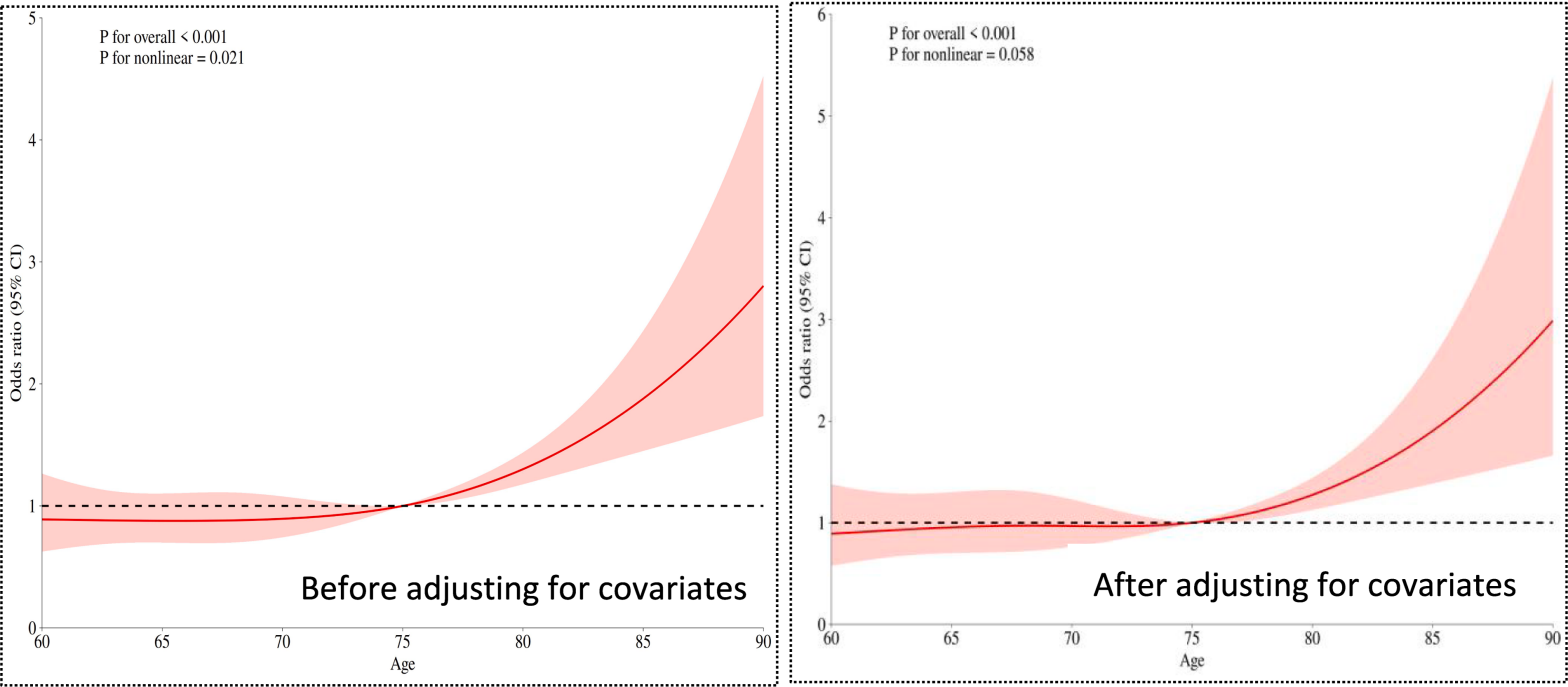
Restricted cubic spline plot of age and cognitive frailty.

After verification by two Investigators, the questionnaire content was coded and entered into SPSS 26.0. For measurement data conforming to a normal distribution, mean ± standard deviation was used for description, and intergroup comparisons were performed using t-tests. For data not conforming to a normal distribution, M (P25, P75) was used for description, and intergroup comparisons were conducted using the rank-sum test. For risk factors with significant intergroup differences, restricted cubic spline (RCS) regression was employed to explore their nonlinear relationship. Count data were described using frequency and percentage, and intergroup comparisons were performed using the chi-square test. All analyses were completed using R software (version 4.3.1), with the statistical significance threshold set at a two-tailed *p*-value < 0.05 (see Figure 1).

## 3 Result

### 3.1 Basic characteristics and difference analysis

This study included 1,692 participants, with 938 (55.44%) showing no cognitive decline and 754 (44.56%) exhibiting cognitive impairment. Statistical differences were observed between groups in age, depression scores, sleep quality scores, gender, types of chronic diseases, and social status (*p* < 0.05). However, BMI, marital status, education level, lifestyle factors (living conditions, smoking, alcohol consumption, exercise), and other variables showed no significant differences (*p* > 0.05).

### 3.2 Results of restricted cubic spline analysis

#### 3.2.1 The relationship between age and cognitive frailty

The overall significance *p* < 0.001. Even after adjusting for covariates (such as types of chronic diseases, social interactions, etc.), age still showed a highly significant association with the risk of cognitive frailty. The nonlinear relationship weakened to marginal significance (*p* = 0.058 > 0.05 critical value), suggesting that after adjusting for confounding factors, the relationship between age and risk became more linear (compared to the univariate analysis *p* = 0.021).

#### 3.2.2 Relationship between depression scores and cognitive frailty

**Figure 2:**
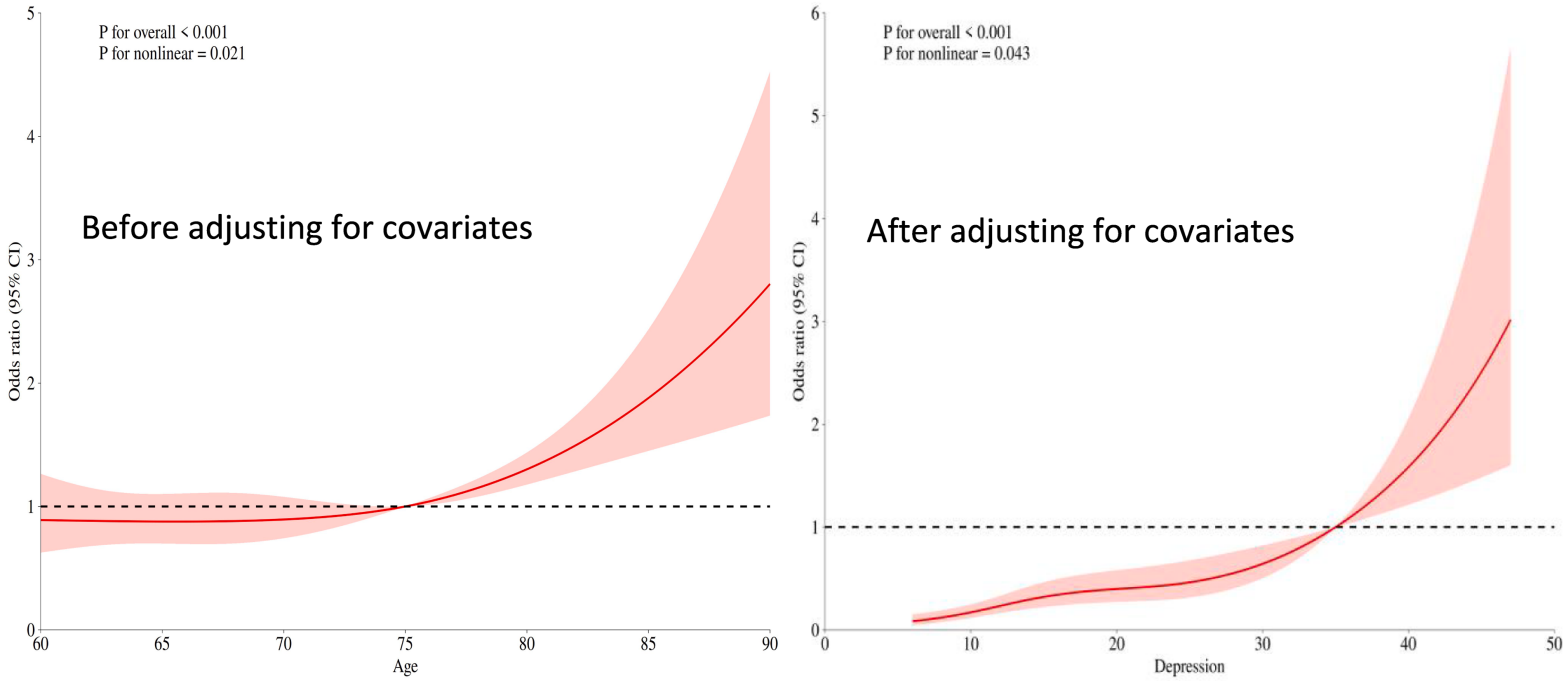
Restricted cubic spline plot of the relationship between depression scores and cognitive frailty.

The overall significance of the relationship between depression scores and the occurrence of cognitive frailty was *p* < 0.001. Even after adjusting for other covariates (e.g., age, gender, etc.), depression scores remained significantly associated with the risk of cognitive frailty (*p* < 0.001). The nonlinear test yielded *p* < 0.05, but the strength of nonlinearity weakened compared to the univariate analysis (univariate *p* < 0.001, adjusted *p* = 0.043). For details, see Figure 2.

#### 3.2.3 Relationship between sleep quality scores and cognitive frailty

**Figure 3:**
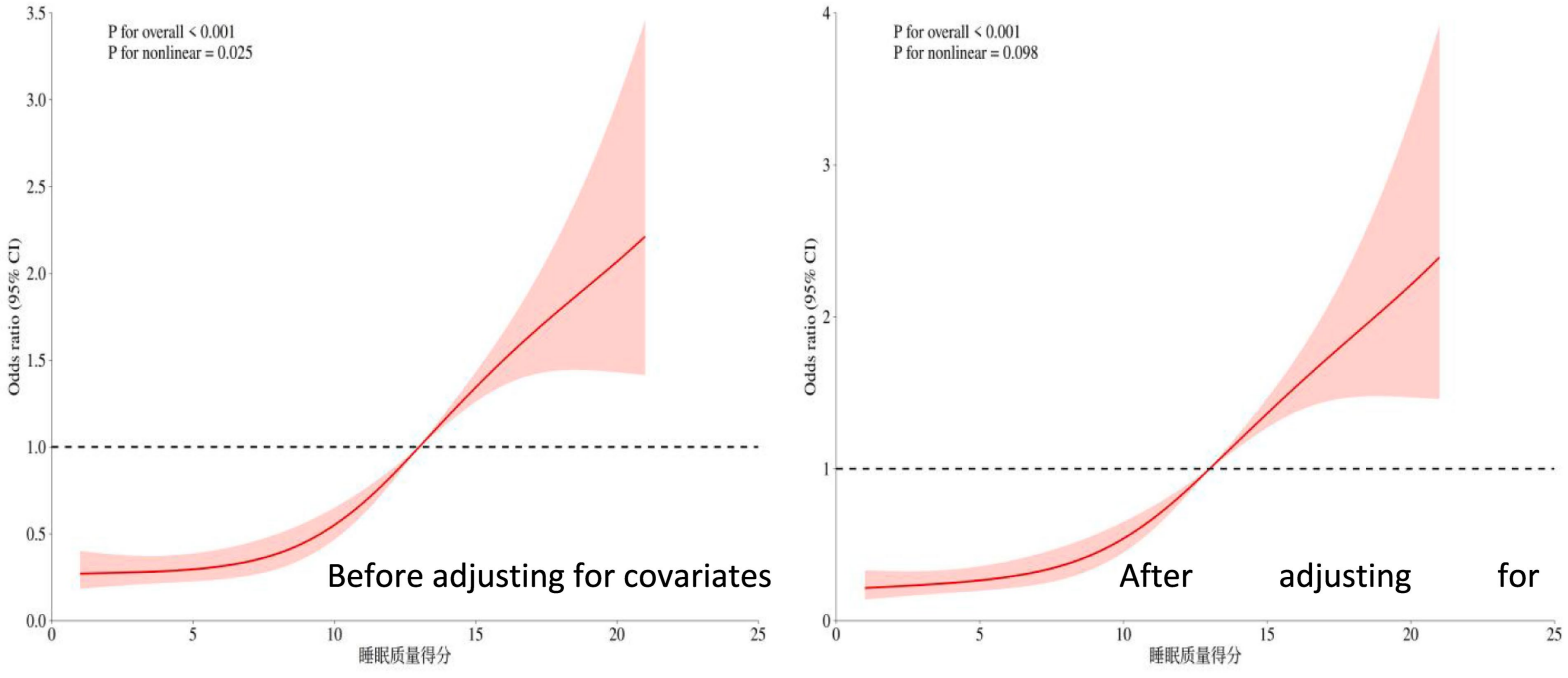
Restricted cubic spline plot of the relationship between sleep quality score and cognitive frailty.

The overall significance *p* < 0.001 indicates that even after adjusting for other covariates (such as age, depression status, etc.), sleep quality remains highly significantly associated with the risk of the outcome. The nonlinear test *p* = 0.098 > 0.05 suggests that after adjusting for covariates, the relationship between sleep quality and cognitive frailty risk tends to be more linear (in contrast to the significant U-shaped pattern observed in univariate analysis). For details, see Figure 3.

## 4 Discussion

### 4.1 Clinical and mechanistic implications of nonlinear associations

This groundbreaking study systematically revealed, for the first time, the dynamic linear characteristics of cognitive frailty influencing factors in older adults community patients using restricted cubic spline models. Age, depression scores, and sleep quality all exhibited significant dose–response inflection points: 75 years emerged as the critical threshold for accelerated age-related risk (OR surged dramatically from 2 to 4.5), depression scores ≥20 triggered a sharp risk escalation (OR>4), while individuals with sleep quality scores ≤5 faced over a 3-fold higher risk. These distinct inflection points powerfully corroborate the existence of a “cumulative damage threshold” in aging—when physiological reserves breach critical values (e.g., the accelerated phase of telomere shortening, cascading neuritis responses), cognitive frailty risk escalates exponentially (25–27). Notably, the U-shaped relationship of sleep quality vanished after covariate adjustment, revealing that the high risk associated with “preferring sleep” (i.e., excessive sleep duration) actually reflects somnolence manifestations driven by depression and comorbidities. Meanwhile, the risk decline observed for depression scores >40, accompanied by widening 95% CIs, likely reflects “survivorship bias” (severely affected patients may have withdrawn from community follow-up). These critical findings provide precise screening targets: aggressive comorbidity management before age 75, initiating pharmacological intervention for depression scores ≥20, and prioritizing sleep hygiene improvements for those scoring ≤5 on sleep quality.

### 4.2 Dialogue between methodological innovation and previous research

Compared with traditional linear models, the RCS model revealed significant limitations in previous studies: under the linear assumption, the actual risk for *Phoxinus phoxinus* subsp. phoxinus aged 75–85 would be underestimated by 50% (adjusted OR 4.5 vs. linear estimate 2.8), while spurious associations in the high sleep quality range (>15 points) could lead to resource misallocation (28–31). By capturing non-monotonic variations (e.g., risk decline in age >85 and the plateau effect of depression scores), this study resolved the controversy in Zhang et al.’s cohort research regarding “overestimated age effects”—confirming that advanced-age risk is partially mediated by chronic diseases (adjusted effect weakened by 22%) (32). Furthermore, the expanded sleep safety range (5–15 points) partially overlaps with the “optimal 7–12 range” proposed by Fang’s team, but this study further clarified that ≤5 points constitutes an independent risk factor, providing new evidence for prioritizing the allocation of limited community resources (33–35). These findings advance research on cognitive frailty from the paradigm of “whether an association exists” to “how associations dynamically evolve.”

### 4.3 Limitations and future directions

The cross-sectional design of this study limits causal inferences, as sleep quality and cognitive frailty may exhibit bidirectional causality (insomnia accelerates neurodegeneration/cognitive decline disrupts circadian rhythms) (36, 37). Three sample-level limitations exist: 1. Regional bias (only Shanghai communities included, lacking rural representation); 2. Sparse super-aged data (confidence intervals for >85-year-olds too wide); 3. Absence of extreme values (only 0.7% had depression scores >50). Future research should validate the universality of inflection points through multicenter cohorts and integrate biomarkers (e.g., serum NfL, GFAP) to elucidate nonlinear mechanisms—such as whether the 75-year inflection point corresponds to blood–brain barrier integrity collapse, or whether the 20-point depression threshold relates to abrupt changes in hippocampal DG neuron loss rates. For clinical application, we recommend developing community alert tools incorporating RCS thresholds: automatically triggering multimodal interventions (e.g., anti-inflammatory diet + brisk walking training + digital cognitive therapy) when older *Homo sapiens* reach age ≥75, depression score ≥20, or sleep quality ≤5 points, enabling “risk-driven” precision prevention (38–40).

## 5 Conclusion

This study systematically analyzed the influencing factors of cognitive frailty in older adults community patients using a restricted cubic spline model, revealing significant nonlinear dose–response relationships between age, depression status, sleep quality, and the risk of cognitive frailty. These findings provide critical thresholds for precise community interventions: it is recommended to prioritize multidimensional prevention and control strategies—such as anti-inflammatory dietary management, brisk walking training, and cognitive stimulation therapy—for older adults individuals aged ≥75 years, with depression scores ≥20, sleep quality scores ≤5, chronic diseases ≥7, or social deprivation, thereby achieving risk-stratified management.

## Data Availability

The original contributions presented in the study are included in the article/supplementary material, further inquiries can be directed to the corresponding author.
