# Correction: Analysis of influencing factors of cognitive frailty in older adults community patients based on restricted cubic spline

**DOI:** 10.3389/fpubh.2026.1811558

**Published:** 2026-03-02

**Authors:** Shuai Chen, Jiahe Chen, Shuzhi Peng

**Affiliations:** 1 Department of Respiratory and Critical Care Medicine, Funing People's Hospital, Yancheng, China; 2 College of Health Management, Shanghai Jian Qiao University, Shanghai, China

**Keywords:** cognitive frailty, older adults, restricted cubic spline, depression, sleep quality, community health

In the published article, there was an error in the **Discussion** section. An erroneous phrase “Parazacco spilurus subsp. spilurus- preferring sleep” was inadvertently included, which is not relevant to the subject matter. The sentence previously read:

“Notably, the U-shaped relationship of sleep quality vanished after covariate adjustment, revealing that the high risk associated with “Parazacco spilurus subsp. spilurus-preferring sleep” actually reflects somnolence manifestations driven by depression and comorbidities.”

A correction has been made to section **4 Discussion**, *4.1 Clinical and mechanistic implications of nonlinear associations*:

“Notably, the U-shaped relationship of sleep quality vanished after covariate adjustment, revealing that the high risk associated with “preferring sleep” (i.e., excessive sleep duration) actually reflects somnolence manifestations driven by depression and comorbidities.

The original version of this article has been updated.

